# Peanut allergy in Italy: A unique Italian perspective

**DOI:** 10.1016/j.jacig.2022.02.001

**Published:** 2022-03-07

**Authors:** Riccardo Asero, Eleonora Nucera, Angela Rizzi, Arianna Aruanno, Carina G. Uasuf, Giuseppina Manzotti, Danilo Villalta, Mariaelisabetta Conte, Elide A. Pastorello, Laura Losappio, Jan V. Schroeder, Elena Pinter, Marzia Miglionico, Lorenzo Vantaggio, Donatella Macchia, Anna Radice, Alessandro M. Marra, Claudio Barzaghi, Annalisa Santucci, Gabriele Cortellini, Silvia Peveri, Marcello Montagni, Antongiulio Demonte, Paolo Borrelli, Micol A. Errico, Federica Rivolta, Valerio Pravettoni, Andrea Sangalli, Monica Magnani, Giorgio Celi, Baoran Yang, Maria T. Costantino, Gaia Deleonardi, Elisa Boni, Marco Gattoni, Fabio Lodi Rizzini, Camilla Di Paolo, Mariacarmela Montera, Annaclaudia Giordano, Marco De Carli, Francesco Murzilli, Federica Fumagalli, Laura Maffeis, Daniele Giovanni Ghiglioni, Simone Centonze, Michela Di Lizia, Paolo Calafiore, Enrico Scala

**Affiliations:** a Ambulatorio di Allergologia, Clinica San Carlo, Paderno Dugnano, Milan, Italy; b Fondazione Policlinico Universitario A. Gemelli IRCCS, Università Cattolica del Sacro Cuore – Roma, Italy; c Allergy Center, Institute of Traslational Pharmacology, National Research Council (CNR), Palermo, Italy; d Servizio di Allergologia, Casa di Cura Beato Palazzolo, Bergamo, Italy; e SSD di Immunologia e Allergologia, PO S. Maria degli Angeli, Pordenone, Italy; f Dipartimento di Allergologia, ASST Grande Ospedale Metropolitano Niguarda, Milano, Italy; g UOC Medicina Interna e Immunologia Clinica, Dipartimento Medicina Traslazionale e di Precisione, Sapienza University of Rome, Rome, Italy; h Allergy and Clinical Immunology Residency Program, Department of Molecular Medicine, Sapienza University of Rome, Italy; i SOS Allergologia e Immunologia Clinica, Ospedale San Giovanni di Dio, Firenze, Italy; j ASST Rhodense - UO Pneumologia - Ospedale di Garbagnate Milanese, Milan, Italy; k ASST Rhodense - Ambulatorio di Allergologia - Ospedale di Rho, Italy; l Ausl Romagna, UOS interdipartimentale di Allergologia, Ospedale degli Infermi di Rimini, Italy; m UOsD Allergologia, Ospedale G. Da Saliceto, Piacenza, Italy; n SSD Dermatologia - Ambulatorio Allergologia e Immunologia Clinica, Ospedale Beauregard, Aosta, Italy; o General Medicin, Immunology and Allergy Department - IRCCS Foundation Ca' Granda Ospedale Maggiore Policlinico - Milan, Italy; p Allergy and Clinical Immunology Residency, University of Milan, Italy; q Ambulatorio di Allergologia DCP AUSL Bologna, Italy; r Centro DH Allergologia e Immunologia Clinica, Ospedale Carlo Poma ASST-Mantova, Italy; s Allergologia e autoimmunità LUM AUSL Bologna, Italy; t Scuola di Specializzazione in Allergologia e Immunologia Clinica Università degli Studi di Bologna, Italy; u SSVD Allergologia Spedali Civili - Università Studi Brescia; v Allergologia e Immunologia Clinica, Ospedale G. Fuscito, Mercato S. Severino; Ospedaliero-Universitaria Ruggi D'Aragona, Salerno, Italy; w S.O.C Medicina 2, Presidio Ospedaliero Santa Maria della Misericordia, Udine, Italy; x U.O.S.D. di Allergologia, Ospedale S.S. Filippo e Nicola, Avezzano, Italy; y Specialista Allergologia ambulatoriale Asl 2, Savona, Italy; z Fondazione IRCCS Ca' Granda Ospedale Maggiore Policlinico di Milano, Pediatric Intermediate Care Unit, Milan, Italy; aa Fondazione IRCCS Ca' Granda Ospedale Maggiore Policlinico di Milano, Pediatric Highly Intensive Care Unit, Milan, Italy; bb Università degli Studi di Milano, Italy; cc U.O.S.D. di Allergologia, P.O. di Giulianova, Italy; dd Istituto Dermopatico dell’Immacolata, Roma, Italy

**Keywords:** Peanut allergy, food allergy, component-resolved diagnosis, lipid transfer protein, seed storage proteins, anaphylaxis

## Abstract

**Background:**

Peanut allergy has not been well characterized in Italy.

**Objective:**

Our aim was to better define the clinical features of peanut allergy in Italy and to detect the peanut proteins involved in allergic reactions.

**Methods:**

A total of 22 centers participated in a prospective survey of peanut allergy over a 6-month period. Clinical histories were confirmed by *in vivo* and/or i*n vitro* diagnostic means in all cases. Potential risk factors for peanut allergy occurrence were considered. Levels of IgE to *Arachis hypogea* (Ara h) 1, 2, 3, 6, 8, and 9 and profilin were measured.

**Results:**

A total of 395 patients (aged 2-80 years) were enrolled. Of the participants, 35% reported local reactions, 38.2% reported systemic reactions, and 26.6% experienced anaphylaxis. The sensitization profile was dominated by Ara h 9 (77% of patients were sensitized to it), whereas 35% were sensitized to pathogenesis-related protein 10 (PR-10) and 26% were sensitized to seed storage proteins (SSPs). Sensitization to 2S albumins (Ara h 2 and Ara h 6) or lipid transfer protein (LTP) was associated with the occurrence of more severe symptoms, whereas profilin and PR-10 sensitization were associated with milder symptoms. Cosensitization to profilin reduced the risk of severe reactions in both Ara h 2– and LTP-sensitized patients. SSP sensitization prevailed in younger patients whereas LTP prevailed in older patients (*P < .*01). SSP sensitization occurred mainly in northern Italy, whereas LTP sensitization prevailed in Italy's center and south. Atopic dermatitis, frequency of peanut ingestion, peanut consumption by other family members, or use of peanut butter did not seem to be risk factors for peanut allergy onset.

**Conclusions:**

In Italy, peanut allergy is rare and dominated by LTP in the country's center and south and by SSP in the north. These 2 sensitizations seem mutually exclusive. The picture differs from that in Anglo-Saxon countries.

Peanut (*Arachis hypogea* [Ara h]) is one of the most frequent causes of food allergy worldwide and is potentially able to cause extremely severe reactions in sensitized individuals.^1^^,^^2^ Peanut allergy is usually lifelong and probably accounts for the majority of fatal allergic events secondary to food. The recent advances in molecular biology have led to identification of an increasing number of peanut allergens, most of which are both heat and pepsin stable and hence potentially able to induce systemic symptoms in subjects with allergy.^3^ Although the relevance of peanut as a food allergen source is unquestioned and supported by a very large number of scientific studies, most of these have, surprisingly enough, been carried out in North America and Northern Europe, where the peanut seed storage proteins (SSPs) (ie, the 2S-albumins Ara h 2 and Ara h 6, the vicilin or 7S-globulin Ara h 1, and the legumin or 11S-globulin Ara h 3, which together are referred to as SSPs) play the major clinical role. In the EuroPrevall study, which is an international study of food allergy in European adults,^4^ peanut sensitization rates showed a high variability between different countries. Interestingly, sensitization to SSPs seems to be strongly associated with an early onset of peanut allergy (ie, before the age of 14 years).^5^ Little is known about this food allergy in Mediterranean countries, in which plant-derived food allergies show several peculiarities owing to the presence of some specific cross-reacting allergens such as lipid transfer protein (LTP) or gibberellin-regulated protein.^6^^,^^7^ In Spain, children and adolescents with peanut allergy show a much lower prevalence of sensitization to peanut SSPs than do children and adolescents in the United States and northern Europe.^8^ Further, Spanish patients show a high prevalence of sensitization to Ara h 9, the peanut LTP.^8^ In Italy, peanut allergy is encountered quite rarely in daily clinical practice, and nothing is known about the relevant offending peanut allergens in patients with allergy. Thus, the Association of Italian Territorial and Hospital Allergologists and Immunologists decided to carry out a multicenter prospective study aimed at developing a better definition of the clinical features of peanut allergy in Italy and detecting the most relevant peanut proteins involved in clinical allergic reactions.

## Methods

A total of 22 Italian allergy centers agreed to participate in the multicenter prospective clinical survey of peanut allergy in the country. In the participating centers, doctors recorded all the cases of unequivocal peanut allergy detected among patients who visited that center between May 15 and November 15, 2021 (6 months), on a standard identical form. The peanut-induced allergic reactions reported by patients included both local reactions (such as oral allergy syndrome or isolated gastrointestinal reaction) and systemic reactions (urticaria/angioedema or anaphylaxis). All clinical histories were confirmed by a positive result of a skin prick test with commercial peanut extract and/or by the detection of IgE specific for peanut extract by ImmunoCAP (Thermo Fisher, Uppsala, Sweden). In the latter case, IgE levels higher than 0.1 kUA/L were considered positive as per the manufacturer’s instructions. The enrolled patients were thoroughly interviewed to detect potential risk factors for peanut allergy, including (1) disease duration, (2) past or current presence of atopic dermatitis, (3) use of peanut butter, (4) frequency of peanut ingestion, and (5) peanut ingestion by other members of the family. The diagnosis of peanut allergy was not confirmed by blinded or open oral food challenge owing to both the severity of several reactions and the fact that many centers were not sufficiently equipped in terms of facilities or personnel to manage possible severe allergic reactions.

### *In vitro* tests

In all enrolled patients the offending peanut allergens were detected by component-resolved diagnosis, measuring specific IgE for Ara h 1, Ara h 2, Ara h 3, Ara h 6, Ara h 8, Ara h 9 (or *Prunus persica* 3 [Pru p 3], the peach LTP), and *Phleum pratense* 12 [Phl p 12], as a marker of profilin reactivity. In this case as well, IgE levels higher than 0.1 kUA/L were considered positive.

### Statistics

The sampled data were recorded and analyzed by using SPSS software (version 27.0.1.0, IBM, Inc, Armonk, NY).

In univariate analysis, the nonparametric Mann-Whitney *U*-test (2 groups) was first used to compare peanut molecules’ continuous IgE levels in subjects with or without a given clinical involvement; then, each variable of interest was dichotomized (as negative or reactive) to study the proportion of subjects with organ involvement in the 2 groups thus obtained. The Pearson chi-square test or Fisher exact test (used for 2 × 2 contingency tables with <50 cases) were used to assess whether paired observations of 2 variables expressed in a contingency table were independent of each other. Finally, we performed multiple logistic regression for the clinical variables with dichotomous scores (present vs absent) to see whether the association between clinical symptoms and reactivity to different peanut allergens was present after simultaneously adjusting for the other variables of interest.

The degree of relationship between the quantitative variables studied was analyzed by using the Pearson correlation (*r*) test, and the most commonly used bivariate correlation technique. A *P* value less than .05 was considered statistically significant.

### Ethical issues

The study was approved by the ethics committee of the coordinating center (Clinica San Carlo) and subsequently by the local ethics committees of all of the other participating centers.

The recruited patients gave informed consent for use of their clinical data in an anonymous form.

## Results

### General findings

The study group consisted of 395 patients (44.1% male and 55.9% female; mean patient age 32 ± 15 years [range 2-80 years]) who were enrolled by the doctors working in the 22 centers participating in the study. Of those patients, 53% (210) came from the central and southern peninsula and the remaining 47% (185) had been recruited in northern Italy. In all, 8% of the patients were younger than 14 years, 25.3% were 14 to 24 years old, 26.9% were 24 to 34 years old, and the remaining 39.6% were older than 35 years. In all, 35% reported local reactions only, 38.2% had experienced systemic reactions, and 26.6% had a history of anaphylactic reactions. No age and sex differences were detected in relation to symptom severity (Table I).

**Table I:** Clinical features of the general study population

Variable	Level	No.	%	Symptoms				ORc	95% CI	*P* value
				Severe		Mild				
				No.	%	No.	%			
Overall		395	100%	256	62%	139	33%			
Sex	Female	221	56%	79	57%	142	55%	1		
	Male	174	44%	60	43%	114	45%	1.1	0.6-1.6	.794
Age	<14 y	32	8%	24	10%	8	6%	1		
	>14 y	359	92%	228	90%	131	94%	0.58	0.2-1.3	.193
Ara h 1 (7S globulin)	No	269	84%	177	82%	92	87%	1		
	Yes	53	16%	39	18%	14	13%	1.44	0.7-2.8	.27
Ara h 2 (2S albumin)	No	275	82%	170	78%	105	90%	1		
	Yes	60	18%	48	22%	12	10%	2.47	1.2-4.8	.007
Ara h 3 (11S globulin)	No	268	88%	173	87%	95	91%	1		
	Yes	36	12%	27	14%	9	9%	1.65	0.7-3.6	.221
Ara h 6 (2S albumin)	No	183	85%	120	82%	63	93%	1		
	Yes	32	15%	27	18%	5	7%	2.283	1.1-7.7	.035
Ara h 8 (PR-10)	No	224	69%	169	78%	55	51%	1		
	Yes	100	31%	47	22%	53	49%	0.289	0.2-0.5	<.001
Ara h 9 (LTP)	No	108	29%	63	26%	45	37%	1		
	Yes	262	71%	184	74%	78	63%	1.68	1.1-2.7	.027
Phl p 12 (profilin)	No	226	82%	171	88%	55	66%	1		
	Yes	51	18%	23	12%	28	34%	0.264	0.1-0.5	<.001

*Phl p*, *Phleum pratense.*

### Sensitization profiles and clinical correlations

**Fig 1:**
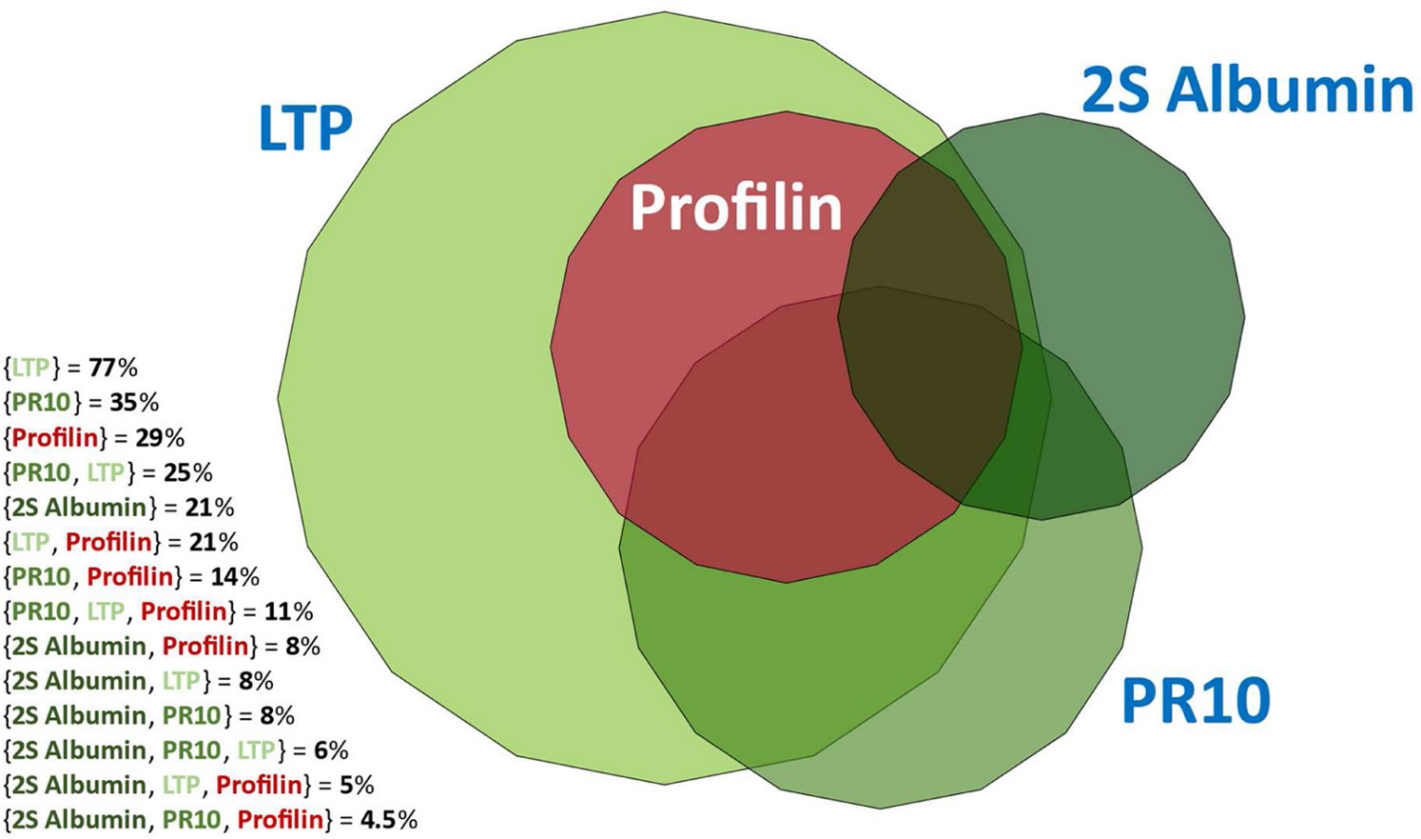
Distribution of peanut sensitization profiles in the study population.

As summarized in Fig 1, the profile of sensitization to peanut molecules was dominated by Ara h 9, with 77% of study participants sensitized to LTP. In all, 35% percent had an allergy to pathogenesis-related protein 10 (PR-10) and 26% had SSP allergy. Interestingly, all patients with profilin allergy (28.7%) were cosensitized to at least 1 other peanut allergen; as a consequence, no study patient was monoreactive to profilin.

As expected, sensitization to 2S albumins (either Ara h 2 or Ara h 6) or LTP was associated with the occurrence of more severe symptoms (Table I). On the other hand, reactivity to 11S or 7S globulins was never associated with an increased risk of severe symptoms. Both profilin and PR-10 sensitization were significantly associated with milder symptoms, and cosensitization to profilin reduced the risk of severe reactions in both Ara h 2–sensitized (*P* = .017) and LTP-sensitized (*P* = .012) patients. Accordingly, multiple logistic regression analysis (when simultaneously adjusting for all the molecules studied, age, and sex) confirmed both the direct relationship between a history of severe reaction and Ara h 9 reactivity (*P* = .006; ORa = 3.331 [95% CI = 1.4-7.8]) and the inverse relationship between severity of symptoms and profilin reactivity (*P* = .03; ORa = 0.394 [95% CI = 0.2-0.9]) or PR-10 (*P* < .001; ORa = 0.272 [95% CI = 0.1-0.6]).

### Age subsets analysis

**Fig 2:**
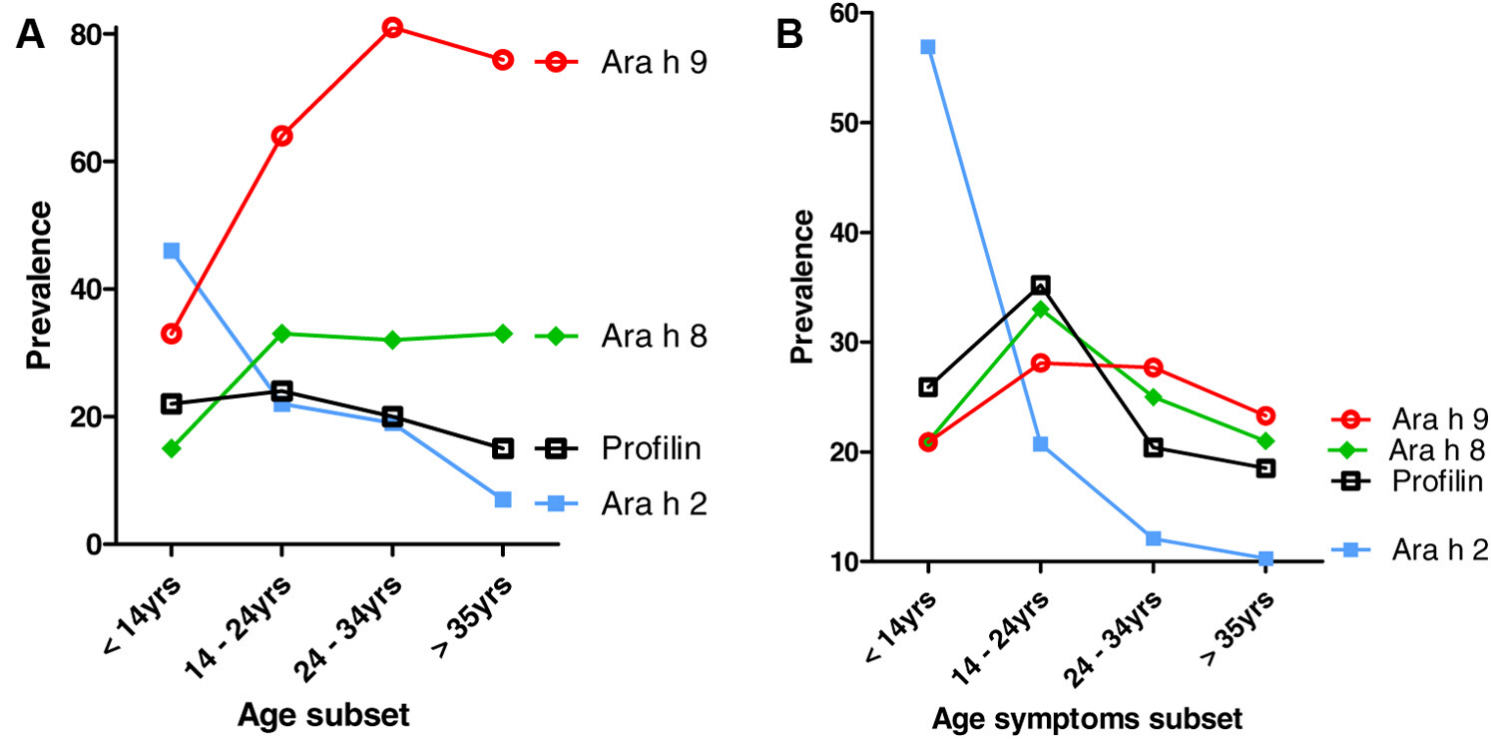
**A,** Prevalence of sensitization to peanut components in the different age subgroups. **B,** Prevalence of sensitization based on age of symptom onset.

To investigate the sensitization profiles as a function of age, the enrolled patients were divided into those younger than 14 years, those aged 14 to 24 years, those aged 25 to 34 years, and those older than 35 years. When the population studied was assessed as a whole (Fig 2, *A*), SSP sensitization was observed more frequently in subjects younger than 14 years (46.9% vs 14.3% of the adult population [*P* < .01]), whereas LTP sensitization behaved in the opposite way, being detected in the adult population in 74.3% of cases versus in 34.4% of cases among the pediatric population (*P* < .01). A similar result was obtained by correlating the prevalence of sensitization with the age of symptom onset obtained by detracting the disease duration from age at the visit (Fig 2, *B*): again, the primary sensitization to Ara h 2 and Ara h 6 occurred with a significantly higher frequency in the pediatric population (*P* < .001), whereas LTP sensitization occurred in mostly in adults.

Despite the absence of a direct association between age and symptom severity in the general population, the analysis of the correlation between specific molecules and the different age subsets considered showed that the reactivity to 2S albumin was significantly (*P* = .01) associated with the risk of severe reaction in patients aged 14 to 24 years (crude odds ratio [ORc] = 5 [95% CI = 1.3-18.8]), whereas LTP reactivity was associated with the risk of severe reaction in the population older than 35 years (*P* = .036; ORc = 2.318 [95% CI = 1.0-5.1]). Both profilin sensitization and PR-10 sensitization were associated with a significant reduction of the risk of severe reaction in patients with peanut allergy who were older than 24 years (*P* < .01), whereas for PR-10 reactivity only, this aspect was observed even in participants younger than 14 years (*P* = .004).

### Geographic differences in peanut sensitization profiles in Italy

On the basis of our previous studies showing a markedly growing prevalence of LTP sensitization in Italy along a north-south gradient,^9^^,^^10^ we analyzed the data as a function of the geographic distribution of patients. As before, the participating study centers were subdivided by location in the northern versus central and southern parts of the country.

A completely different peanut allergen reactivity profile was recorded in the different geographic areas of the country. In fact, whereas 77.6 of 100 patients in the center and south were reactive to the LTP Ara h 9 (a prevalence that was significantly higher than the prevalence of 62.7% observed in the north [*P* < .01]), the prevalence of sensitization to the other components studied was always higher in the north (for PR-10 sensitization, 46.9% vs 18.2% [*P* < .01]; for profilin sensitization, 26.5% vs 14 % [*P* < .01]; and for 2S albumin sensitization, 26.9% vs 9.7% [*P* < .01]). LTP sensitization and SSP sensitization appeared to be mutually exclusive, as only 15 patients showed both types of sensitization.

Interestingly, the occurrence of severe reactions after peanut intake was correlated with 2S albumin allergy in northern Italy (ORc = 3.022 [95% CI = 1.3-6.8]; *P* = .007) and with LTP sensitization in Italy's center and south (ORc = 3.188 95% CI = 1.6-6.3]; *P* = .001). Sensitization to profilin was associated with a significant reduction in the risk of severe reaction both in the north (ORc = 0.286 95% CI = 0.1-0.7]; *P* = .008) and in the center and south (ORc = 0.226; [95% CI = 0.1-0.5]; *P* < .001), whereas reactivity to PR-10 exerted a protective role only in those patients with peanut allergy who had been recruited in the central and southern parts of the country (ORc = 0.112 [95% = CI = 0.05-0.3]; *P* < .001).

### Detection of risk factors for primary peanut sensitization

We were unable to detect any relevance as a risk factor for primary sensitization to specific peanut allergens when we analyzed past or current presence of atopic dermatitis, frequency of peanut ingestion, or peanut consumption by other family members, as well as the use of peanut butter. However, regarding peanut butter, it has to be considered that this food was used very rarely in the study population, although the rate of peanut butter use was 2 times higher in the northern part of Italy than in the center and south, in parallel with the prevalence of SSP sensitization.

## Discussion

This was the first multicenter study of peanut allergy carried out in Italy. We believe that several interesting aspects emerged. First, peanut allergy is rather rare in Italy. In fact, although this was not an epidemiologic study, it took 6 months for the 22 participating allergy centers to identify the 395 study subjects. An approximate estimate of the prevalence was calculated by sampling the data coming from 4 randomly selected participating centers that provided 50 cases of peanut allergy among about 6900 visits carried out in 6 months, corresponding to a prevalence of 0.7%. No difference was detected between adult-oriented centers (in which it was detected in 42 of 5700 patients [0.7%]) and the pediatric center (in which it was detected in 8 of 1200 patients [0.67%]; notably, 7 of 8 of these patients had SSP allergy). Of course, because these 6900 patients represent only a biased fraction of the general population, it is possible to infer that a realistic prevalence of peanut allergy in Italy is much lower than 0.7% in these areas.

Another interesting aspect is that most cases of peanut allergy or sensitization were secondary to the sensitization to pollen allergens (PR-10 or profilin) and/or to peach LTP, which most probably constitute the primary sensitizers. Thus, primary sensitization to peanut, as diagnosed by IgE reactivity to the SSPs, Ara h 1, Ara h 2 and/or Ara h 6, and Ara h 3, represents a rather rare event. The reasons for this reduced incidence are unclear; we were unable to detect any significant risk factor for the sensitization to primary peanut allergens among the factors frequency of peanut ingestion, frequency of peanut ingestion in the family, atopic dermatitis, and use of peanut butter. Regarding this latter point, our analysis was probably hampered by the very uncommon use of peanut butter in the Italian cuisine. The fact remains that the use of peanut butter was 2 times higher in the northern region of Italy than in the central and southern regions, thus paralleling the prevalence of peanut SSP sensitization.

Another interesting aspect is that this study clearly showed that cosensitization to stable (SSP and/or LTP) and labile (PR-10 and profilin) peanut allergens leads to a significantly reduced severity of allergic reactions following the ingestion of peanuts. In other words, sensitization to labile food allergens appears to reduce the risk of severe reactions induced by stable allergens. How IgE specific for labile allergens of a certain food (which are in most cases unable to reach the gastrointestinal tract in an unmodified form) succeeds in reducing the severity of the clinical reactions induced by the stable allergens of the same food remains unclear. One possibility might be reduction of the density of SSP- and LTP-specific IgE on effector cells' surface, leading them to a less pronounced degranulation. Another possibility might be that IgE to labile allergens (which notably, are cross-reactive to pollen allergens) shows a higher affinity for IgE receptors on effector cells, thus emerging as the “winner” in the competition with IgE to stable allergens for receptor binding. In any case, there is an evident need for further studies to clarify this point. Nonetheless, the effect is clear and has been confirmed in different studies by our group^11^ and by other groups as well.^12^

The analysis of the geographic distribution of peanut allergen sensitization revealed several interesting aspects of this kind of food allergy in Italy. By comparing our data with those of the only previous international study of peanut allergy carried out in 3 distinct geographic regions,^8^ namely, Spain (which is characterized by a high prevalence of LTP hypersensitivity and PR-10 sensitization is virtually absent), Sweden (which is characterized by a high prevalence of PR-10 sensitization, high prevalence of sensitization to SSP, and absence of LTP sensitization), and the United States (which is characterized by very high prevalence of sensitization to SSP and low prevalence of both PR-10 and LTP sensitization), it turned out that the picture in the central and southern regions Italy is very similar to that found in Spain but it changes northward, where the distribution of the sensitizations starts gradually resembling that in northern Europe more (although a significant prevalence of LTP sensitization remains). In no part of Italy does the sensitization profile resemble that found in the United States. The reasons why the distribution of LTP and peanut SSP sensitizations change with latitude are unclear. Italy is very much a composite country, encompassing continental climate regions in the north as well as Mediterranean climate regions in its central and southern regions, distributed along 1500 kilometers in the north-south direction. We had already detected marked changes in the prevalence of LTP allergy in this country in 2009.^9^ What is new in the present study is the observation of a quite similar but opposite picture for peanut SSP sensitization. Again, the reasons why patients living in northern Italy (and Europe as well) are more prone to become sensitized to peanut SSPs remain unclear. Nonetheless, these geographic variations suggest a heavy influence of hitherto unidentified environmental risk factors, and not only of the dietary habits. Regarding the putative influence of peanut butter consumption on peanut sensitization, a study carried out in Italy will never obtain sufficient statistical power to respond to this question. A study carried out in a country in which peanut butter is commonly used is needed.

In keeping with the existing literature, SSP sensitization was observed more frequently in the population younger than 14 years, in which it was significantly associated with an increased risk of serious adverse reactions after the ingestion of peanuts. Reactivity to LTP behaved in the opposite way: it was more frequent in adults, and it constituted the main cause of severe peanut-induced allergic reaction in this age class significantly more than SSP did. A similar trend was observed with PR-10 sensitization. This latter finding confirms the markedly higher prevalence of *de novo* sensitization to birch pollen in adult subjects, which was already detected in northern Italy several years ago.^13^ The reasons why peanut SSP sensitization prevails markedly in younger subjects are unclear. There are 2 possible explanations for this fact. The first is that peanut SSP allergy might behave similarly to hen’s egg or cow’s milk allergy in children, vanishing gradually in the large majority of cases.^14^^,^^15^ However, although peanut allergy may resolve in some cases,^16^ this does not seem to be the case in the majority of patients, in whom peanut allergy seems to persist until adulthood.^17^ The second explanation is that peanut allergy due to SSP sensitization might have started to increase in Italy only recently and occurs mostly at a young age. A follow-up study is needed to clarify this point.

Finally, the subanalysis of the clinical significance of sensitization to the different peanut SSPs clearly confirmed the markedly higher relevance of sensitization to the 2S-albumins, Ara h 2, and Ara h 6 over the peanut legumin and vicilin, Ara h 1 and Ara h 3, in terms of clinical reactivity.^18^

The main conclusion of the present study is that in Italy, severe peanut allergy shows 2 different and mutually exclusive causes: SSP in the pediatric population and LTP in the adult population. Reactivity to profilins appears to protect against the risk of severe reactions to peanuts.
